# Association between lactate-to-albumin ratio and 28-days all-cause mortality in patients with sepsis-associated liver injury: a retrospective cohort study

**DOI:** 10.1186/s12879-024-08978-x

**Published:** 2024-01-09

**Authors:** Xiaona Yi, Dongcai Jin, Shanshan Huang, Zhenye Xie, Meixia Zheng, Fen Zhou, Yuhong Jin

**Affiliations:** Department of Critical Care Medicine, Ningbo Medical Center Lihuili Hospital, Ningbo, Zhejiang China

**Keywords:** Lactate-to-albumin ratio, Liver injury, All-cause mortality, 28-days, Sepsis, MIMIC IV

## Abstract

**Background:**

The mortality rate of sepsis-associated liver injury (SALI) is relatively high, but there is currently no authoritative prognostic criterion for the outcome of SALI. Meanwhile, lactate-to-albumin ratio (LAR) has been confirmed to be associated with mortality rates in conditions such as sepsis, heart failure, and respiratory failure. However, there is a scarcity of research reporting on the association between LAR and SALI. This study aimed to elucidate the association between LAR and the 28-day mortality rate of SALI.

**Methods:**

In this retrospective cohort study, data were obtained from the Medical Information Mart for Intensive Care IV (v2.2). Adult patients with SALI were admitted to the intensive care unit in this study. The LAR level at admission was included, and the primary aim was to assess the relationship between the LAR and 28-day all-cause mortality.

**Results:**

A total of 341 patients with SALI (SALI) were screened. They were divided into a survival group (241) and a non-survival group (100), and the 28-day mortality rate was 29.3%. Multivariable Cox regression analysis revealed that for every 1-unit increase in LAR, the 28-day mortality risk for SALI patients increased by 21%, with an HR of 1.21 (95% CI 1.11 ~ 1.31, *p* < 0.001).

**Conclusions:**

This study indicates that in patients with SALI, a higher LAR is associated with an increased risk of all-cause mortality within 28 days of admission. This suggests that LAR may serve as an independent risk factor for adverse outcomes in SALI patients.

**Supplementary Information:**

The online version contains supplementary material available at 10.1186/s12879-024-08978-x.

## Introduction

Sepsis is a global public health challenge characterized by a dysregulated host response to infection, resulting in life-threatening organ dysfunction [1, 2]. It affects millions of individuals worldwide annually, with septic shock and sepsis-related mortality rates of one in three and one in six, respectively [3, 4]. Sepsis represents an intricate and dysregulated host reaction to infection, potentially culminating in multiple organ dysfunction syndrome (MODS) [5]. Due to its critical involvement in metabolism, synthesis, and immune pathways, the liver plays a key role in sepsis-associated liver injury (SALI), which assumes a crucial role in the complications of sepsis. In the context of sepsis, the liver experiences multifaceted alterations, encompassing changes in immune responses, metabolic processes, microvascular dynamics, and coagulation, ultimately resulting in hepatic dysfunction [6]. We recognize that the severity of SALI is significant. As a result, guidelines and studies have emerged, diagnosing SALI in patients based on total bilirubin and INR [7]. However, alternative literature defines this condition using higher levels of total bilirubin and alanine transaminase [8]. Early recognition and appropriate management of SALI are crucial for preventing associated mortality. Thus, a comprehensive understanding of the involvement of the liver in sepsis can help mitigate SALI and improve patient outcomes.

Lactate is produced by anaerobic glycolysis. It is well known that hyperlactatemia is an established indicator of tissue and organ hypoxia [9]. Elevated lactate levels have been associated with higher mortality rates in ICU patients [10]. The liver plays a vital role in lactic acid metabolism. Therefore, abnormal liver function affects lactic acid levels, such as acid–base imbalance and lactic acidosis in liver cirrhosis. In patients with chronic liver diseases (e.g., cirrhosis), lactic acid levels do not accurately reflect tissue hypoxia [11]. Similarly, metformin may also affect the body's lactic acid level [12]. A single lactic acid level cannot fully reflect liver function. Albumin is synthesized in the liver and is correlated with the inflammatory state. With decreasing serum albumin, the mortality of critically ill patients increases substantially [13]. However, malnutrition and chronic inflammation may also affect patient albumin levels. In this regard, predictions based solely on albumin levels are also subject to limitations [14]. Recent research has demonstrated the predictive value of Lactate-to-Albumin Ratio (LAR) in sepsis [15] and septic shock [16]. However, its association with the 28-day mortality in patients with SALI remains unclear.

This study aimed to investigate the relationship between LAR and all-cause mortality within 28 days after admission in patients with SALI.

## Material and methods

### Data source

This retrospective cohort study was conducted in accordance with the STROBE guidelines [17]. Our study utilized deidentified data from the MIMIC-IV (v2.2) database at the Massachusetts Institute of Technology (MIT). The database is continually being updated, and we utilized the latest version, Version 2.2, released on January 6, 2023. Patients with SALI (SALI) admitted to the ICU of the Beth Israel Deaconess Medical Center in Boston between 2008 and 2019 were included. Because the data came from the public database and is de-identified, the Institutional Review Board of the Massachusetts Institute of Technology and Beth Israel Deaconess Medical Center allows exemption of patients from the requirement of informed consent [18]. The first author, Xiaona Yi, completed the training on “Data or Specimens Only Research” and was allowed to use the public data (ID: 52,390,976). This study conformed to the provisions of the Declaration of Helsinki (revised in 2013). The Medical Ethics Committee of the Ningbo Medical Center Lihuili Hospital agreed to waive the ethics review because anonymous data were used in this study (ID: KY2023ML011).

### Population selection criteria

Among 432,231 admissions in the MIMIC-IV database, 73,181 were ICU admissions. In total, 50,920 patients were admitted to the ICU for the first time [19]. According to the guidelines of the Surviving Sepsis Campaign [20], a total bilirubin level > 2 mg/dL (34.2 μmol/L) and INR > 1.5 in sepsis patients are diagnostic criteria for SALI. This diagnostic criterion has been cited in multiple publications.

The inclusion criteria of this study are: 1) adults over 18 years old who are hospitalized for the first time and admitted to ICU for the first time in the MIMICIV database; 2) Meet the diagnosis of sepsis3 or increased by SOFA ≥ 2; 3) Meet the diagnostic criteria of SALI.

The following patients were excluded after further screening: (1) lack of 24-h blood lactate and serum albumin measurements; (2) ICU stay of < 24 h; (3) admissions related to liver disease, alcoholism, cirrhosis, autoimmune hepatitis, or malignant liver tumors.

### Data source and data collection

In this study, the LAR was chosen as the primary variable. We specifically selected lactate and albumin measurements obtained on the first day after admission to mitigate the potential confounding effects of therapeutic interventions.

The initial baseline parameters were collected using Structure Query Language (SQL), PostgreSQL tools (version 13.0), and Navicat Premium 16 on the first day following ICU admission. These parameters encompassed various factors such as demographics, vital signs, comorbidities, and assessment scale scores (Simplified Acute Physiology Score II, Sequential Organ Failure Assessment), the first measured laboratory results at the time of admission to the ICU, during ICU admission. In addition, the use of vasoactive agents, continuous renal replacement therapy (CRRT), and mechanical ventilation was also extracted.

### Statistical analysis

In the description of population characteristics, if the continuous variables conformed to a normal distribution, the student’s t-test method was used to express them with mean ± standard deviation. The Mann–Whitney U test method was applied to the non-normal distribution data of continuous variables, which were expressed as the mean (95% CIs). If it was a classification variable, it was compared using the χ ^2^ test and expressed as percentage frequency (%). The missing data is illustrated in Supplementary Table 1. We employed a multiple imputation method, generating five imputed datasets for COX regression analysis. Statistical significance was defined as *p* < 0.05. The results of the multiple imputation method were consistent with those of the main analysis (Supplementary Table 2).

To analyze categorical variables according to the LAR quartiles [21] (0.77,1.50,3), one-way analyses or Kruskal–Wallis tests were used. In our study, HR and 95% confidence interval were calculated using multivariate COX regression analysis to evaluate the relationship between LAR and SALI. LAR was categorized into quartiles and its association with SALI was examined using Cox regression analysis.

The covariates whose initial regression coefficients change by more than 10% are selected according to the previous reports and the incremental increase or elimination of the covariates in the basic model and the complete model. The variance spreading factor (VIF) was used to determine whether a collinear relationship existed. VIF > 2 indicates a collinear relationship. Model 1 was adjusted for age and sex; Model 2 included additional covariates such as sex, age, MBP, and SpO2, while Model 3 was further adjusted for sex, age, MBP, SpO2, BUN, RDW, PTT, and Vasoactive agent. Cox multivariate regression analysis and trend tests were performed for LAR quartiles.

We conducted a restricted cubic spline analysis to investigate the potential nonlinear relationship between LAR and the 28-day mortality rate in patients with SALI. A forest plot was generated to illustrate subgroup analyses and assess the interaction effects between LAR and 28-day mortality in SALI patients. The relationship between LAR quartiles and the 28-day mortality rate was assessed using a Kaplan–Meier (KM) survival curve. Furthermore, we performed a Cox multivariate regression analysis based on different diagnostic criteria for SALI to demonstrate the robustness of our results.

The data analysis process was performed using packages R4.1.2 (The R Foundation1) software and Free Statistics software version 1.8. Statistical significance was defined as *p* < 0.05.

## Results

### Study population and baseline characteristics

**Fig. 1:**
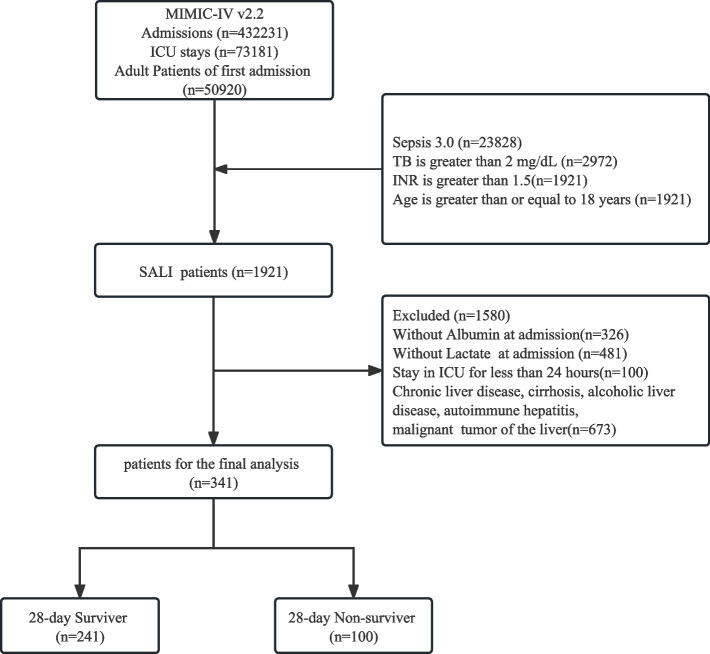
Flowchart screening for eligibility for the study. MIMIC, Medical Information Mart for Intensive Care; SALI, SALI

This study ultimately included 341 cases of sepsis-related liver injury patients, and the flowchart of the patient inclusion process is illustrated in Fig. 1**.**

The baseline characteristics of patients with SALI and their 28-day survival rate are presented in Table 1. The mean age of participants was 62(44,75) years, with 41.3% being female. Within the SALI cohort, 29.3% of the patients experienced all-cause mortality within 28 days. There were statistically significant differences in the SAPS II score, SOFA score, and Charlson comorbidity index between the non-survivor and survivor groups. Statistically significant differences (*P*-value < 0.05) were found in various laboratory parameters, including RDW, BUN, AG, bicarbonate, creatinine, potassium, INR, PT, PTT, AST, LDL, PH, PO2, and base excess. The non-survivor group also exhibited higher rates of mechanical ventilation, CRRT, and vasoactive agent usage, as well as shorter hospital and ICU stays, all of which demonstrated statistical significance (*P* < 0.05).

**Table 1:** Baseline characteristics of 28-day all-cause mortality

Variables	28-day all-cause mortality		*p*
	Survivors (*n* = 241)	Non-survivors (*n* = 100)	
**Baseline characteristics**			
Sex, n (%)			0.745
Male	140 (58.1)	60 (60)	
Female	101 (41.9)	40 (40)	
Age (year)	62.0 (44.0, 75.0)	66.5 (54.8, 76.0)	0.097
Ethnicity (%)			0.154
White	162 (67.2)	57 (57)	
Black/Africa American	17 (7.1)	7 (7)	
Other	62 (25.7)	36 (36)	
Admission, n (%)			0.325
Emergence	131 (54.4)	49 (49)	
Urgent	75 (31.1)	40 (40)	
Surgical	9 (3.7)	1 (1)	
Observation	19 (7.9)	9 (9)	
Elective	7 (2.9)	1 (1)	
Insurance, n (%)			0.04
Medicaid	26 (10.8)	6 (6)	
Medicare	91 (37.8)	52 (52)	
Other	124 (51.5)	42 (42)	
BMI (kg/m^2^)	27.7 (25.3, 32.2)	28.8 (25.7, 33.8)	0.333
Los in hospital(day)	16.2 (9.9, 27.0)	5.6 (2.3, 13.0)	< 0.001
Los in ICU (day)	5.7 (3.0, 11.2)	4.1 (2.3, 9.3)	0.015
LAR	1.4 (0.7, 2.5)	2.2 (1.1, 3.9)	< 0.001
**Vital signs**			
HR (bpm)	114.5 ± 22.1	121.8 ± 25.6	0.008
SBP (mmHg)	85.0 (76.5, 92.0)	76.0 (65.0, 83.0)	< 0.001
DBP (mmHg)	44.0 ± 11.0	39.0 ± 12.3	< 0.001
MBP (mmHg)	55.0 (48.0, 63.0)	50.5 (39.2, 60.0)	0.004
RR (Bpm)	30.0 (25.0, 34.0)	30.0 (27.0, 35.0)	0.344
T (℃)	37.4 (37.1, 38.2)	37.4 (37.0, 37.9)	0.532
SpO2 (%)	92.0 (90.0, 95.0)	91.0 (84.5, 94.0)	< 0.001
**Laboratory tests**			
WBC (10^9^)	9.6 (5.7, 14.7)	11.5 (6.5, 16.1)	0.15
Platelets (10^9^)	108.0 (67.0, 174.0)	92.0 (53.8, 161.0)	0.188
RBC (10^12^)	3.2 (2.8, 3.6)	3.2 (2.7, 4.0)	0.667
RDW (%)	15.8 (14.5, 17.9)	17.0 (15.2, 18.6)	0.017
HCT (%)	28.9 ± 7.9	28.9 ± 7.9	0.98
Hb(g/dL)	9.6 ± 2.6	9.5 ± 2.6	0.616
ALB(g/dL)	2.8 ± 0.6	2.7 ± 0.7	0.058
AG (m Eq/L)	19.0 (15.0, 24.0)	23.0 (19.8, 28.2)	< 0.001
Bicarbonate(mmol/L)	18.0 (13.0, 21.0)	16.0 (12.0, 20.0)	0.008
BUN (mg/dL)	22.0 (15.0, 33.0)	31.0 (20.5, 51.2)	< 0.001
Calcium(mmol/L)	7.5 (6.9, 8.1)	7.5 (6.9, 7.9)	0.604
Cr (u mol/L)	1.6 (1.1, 2.5)	2.3 (1.5, 3.8)	< 0.001
Glucose(mg/dL)	158.0 (124.0, 220.0)	176.0 (131.0, 243.5)	0.151
Sodium(mmol/L)	141.0 (137.0, 143.0)	141.0 (138.0, 146.0)	0.132
Potassium(mmol/L)	4.6 (4.1, 5.2)	5.0 (4.6, 5.5)	< 0.001
Fibrinogen(g/L)	190.0 (115.0, 367.0)	188.0 (126.0, 336.0)	0.797
INR	2.1 (1.8, 2.9)	2.6 (1.9, 4.6)	< 0.001
PT (s)	22.4 (19.1, 30.3)	27.9 (21.0, 49.2)	< 0.001
PTT(s)	43.0 (35.2, 62.6)	54.2 (41.5, 78.8)	0.002
ALT(U/L)	191.0 (43.0, 804.0)	409.0 (51.2, 1278.2)	0.093
ALP(U/L)	120.0 (71.0, 194.0)	128.5 (85.8, 200.0)	0.225
AST(U/L)	230.0 (77.0, 1189.0)	679.5 (146.2, 1928.8)	0.004
TB (u mol/L)	3.7 (2.7, 5.5)	3.6 (2.6, 6.2)	0.815
LDL(U/L)	461.0 (274.0, 1240.8)	984.0 (499.0, 2782.5)	< 0.001
Lactate(mmol/L)	3.7 (2.2, 6.8)	6.4 (3.3, 9.8)	< 0.001
pH	7.3 (7.2, 7.4)	7.2 (7.1, 7.3)	0.007
PO_2_(mmHg)	73.0 (59.0, 95.0)	65.5 (52.0, 89.2)	0.025
PCO_2_(mmHg)	43.0 (36.0, 51.0)	46.0 (37.0, 53.0)	0.327
BE	-7.0 (-11.0, -3.0)	-9.0 (-14.0, -4.8)	0.004
**Scoring systems**			
SAPSII	48.4 ± 16.3	60.8 ± 15.6	< 0.001
SOFA	5.0 (3.0, 7.0)	7.0 (4.0, 9.0)	0.011
**Interventions**			
Vasoactive agent, n (%)	177 (73.4)	86 (86)	0.012
CRRT, n (%)	45 (18.7)	37 (37)	< 0.001
Ventilation, n (%)	142 (58.9)	74 (74)	0.009
**Infection site, n (%)**			
Abdominal infection, n (%)	22 (9.1)	4 (4)	0.104
Pneumonia, n (%)	78 (32.4)	25 (25)	0.177
Urinary infection, n (%)	36 (14.9)	16 (16)	0.804
SSTI, n (%)	3 (1.2)	2 (2)	0.633
**Comorbidities**			
CCI	5.0 (3.0, 7.0)	6.0 (4.0, 8.0)	0.01
MI, n (%)	23 (9.5)	27 (27)	< 0.001
CHF, n (%)	77 (32)	34 (34)	0.713
CPD, n (%)	52 (21.6)	19 (19)	0.594
Diabetes, n (%)	56 (23.2)	25 (25)	0.728
Renal disease, n (%)	45 (18.7)	21 (21)	0.62

*T* Temperature, *HR* heart rate, *RR* Respiratory rate, *CCI* charlson comorbidity index, *SpO2* Peripheral capillary oxygen saturation, *SBP* systolic blood pressure, *DBP* Diastolic blood pressure, *MBP* mean arterial pressure, *SAPS II* Simplified Acute Physiology Score II, *SOFA* Sequential Organ Failure Assessment, *HCT* hematocrit, *HB* hemoglobin, *RBC* red blood cell counts, *RDW* Red blood cell distribution width, *WBC* white blood cell counts, *ALB* albumin, *BE* base excess, *TB* Bilirubin total, *AG* Anion gap, *Cr* creatinine, *BUN* blood urine nitrogen, *ALT* Alanine transaminase, *AST* Aspartate Transaminase, *ALP* Alkaline phosphatase; *LDL* Low-Density Lipoprotein, *INR* International normalized ratio, *PT* Prothrombin time, *PTT* Activated partial thromboplastin time, *CRRT* Continuous renal replacement therapy, *CHF* Congestive heart failure, *CPD* Chronic pulmonary disease, *MI* Myocardial infarct, *RD* renal disease, *SSTI* Skin and soft tissues infection

### LAR is an independent risk factor for 28-day mortality of SALI

LAR was identified as an independent correlate of the 28-day all-cause mortality rate in SALI patients. Multivariable Cox regression analysis revealed that in the initial model, the Hazard Ratio (HR) for the association between LAR and the 28-day mortality rate was 1.23 (95% CI: 1.14–1.33, *p* < 0.001). In the fully adjusted model, the HR remained significant at 1.21 (95% CI: 1.11–1.31, *p* < 0.001). The quartile-stratified analysis corroborated that, in comparison to the lowest quartile, the group within the highest quartile exhibited a Hazard Ratio (HR) of 4.25 (95% CI: 2.1–8.6, *p* < 0.001), with a trend test p-value below 0.001(Table 2).

**Table 2:** Multivariate COX analysis between LAR level and 28-day mortality

Variable	total	event_%	Non-adjusted		MODEL1		MODEL2		MODEL3	
			HR (95%CI)	*P* value	HR (95%CI)	*P* value	HR (95%CI)	*P* value	HR (95%CI)	*P* value
LAR	341	100 (29.3)	1.23 (1.14 ~ 1.33)	< 0.001	1.24 (1.15 ~ 1.34)	< 0.001	1.19 (1.1 ~ 1.29)	< 0.001	1.21 (1.11 ~ 1.31)	< 0.001
LAR.Q										
LAR.Q1(≤ 0.76)	82	14 (17.1)	1(Ref)		1(Ref)		1(Ref)		1(Ref)	
LAR.Q2(0.77–1.49)	88	22 (25)	1.57 (0.8 ~ 3.07)	0.186	1.57 (0.8 ~ 3.08)	0.185	1.54 (0.79 ~ 3.02)	0.207	1.95 (0.96 ~ 3.97)	0.065
LAR.Q3(1.50–3)	85	23 (27.1)	1.78 (0.91 ~ 3.45)	0.09	1.79 (0.92 ~ 3.48)	0.088	1.64 (0.83 ~ 3.21)	0.153	2.25 (1.06 ~ 4.75)	0.034
LAR.Q4(> 3)	86	41 (47.7)	3.63 (1.98 ~ 6.67)	< 0.001	3.79 (2.06 ~ 6.96)	< 0.001	3.04 (1.62 ~ 5.7)	0.001	4.25 (2.1 ~ 8.6)	< 0.001
P for trend				< 0.001		< 0.001		< 0.001		< 0.001

MODEL 1: sex, age

MODEL 2: sex, age, *MBP*, *SpO*_*2*_

MODEL 3: sex, age, *MBP*, *SpO2*, *BUN*, *RDW*, *PTT*, Vasoactive agent

We applied a restricted cubic spline to illustrate the fitted curve, indicating that the relationship between LAR and 28-day mortality, upon full adjustment, is not nonlinear (P for nonlinearity = 0.123) (Supplementary Figure).

**Fig. 2:**
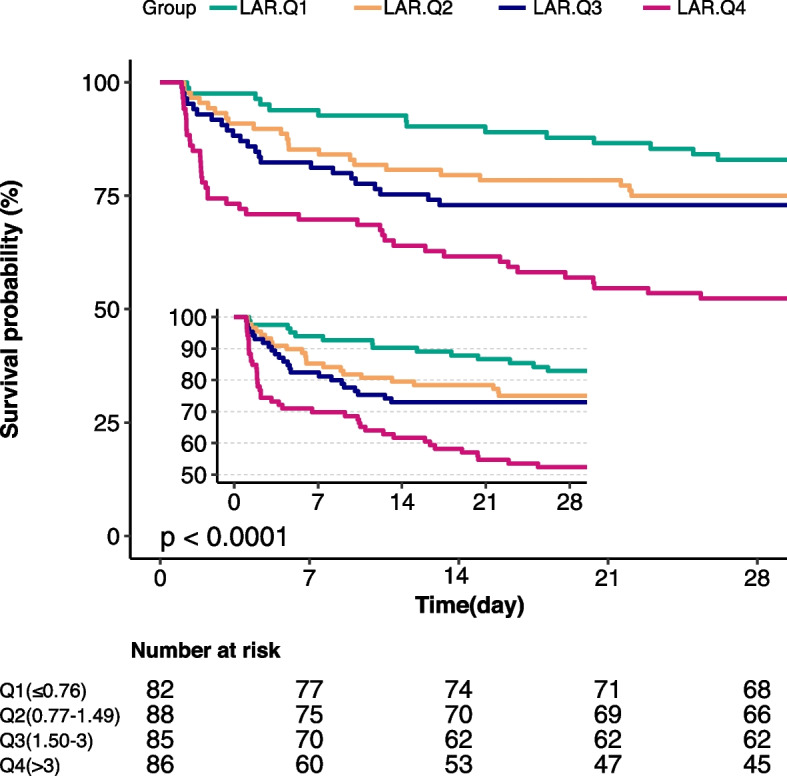
Kaplan–Meier survival analysis curves for all-cause mortality within 28-day of ICU admission. The LAR was grouped according to the quartile, the survival curve of the four groups is shown in the figure, and the shaded area represents the 95% confidence interval

The quartile-stratified Kaplan–Meier curves demonstrate the lowest survival rate in the highest quartile group, with a significant statistical difference observed (*P* < 0.001, Fig. 2). This further reaffirms the robustness of our study findings.

At the same time, we conducted statistical analysis based on different diagnostic criteria of SALI (total bilirubin > 4 umol/L and alanine aminotransferase (ALT) greater than twice the upper limit). The multivariate Cox regression analysis in the fully adjusted model unveiled a hazard ratio of 1.49 (95% CI 1.13–1.96, *p* = 0.004). When stratifying LAR into quartiles, compared to the lowest quartile group, the highest quartile group exhibited a substantially elevated hazard ratio (HR) of 47.82 (95% CI 3.6–634.58, *p* = 0.003) (Supplementary Table 3). This analysis affirms the resilience of our research findings regarding the association between LAR and 28-day mortality in patients with SALI.

### Subgroup analysis

**Fig. 3:**
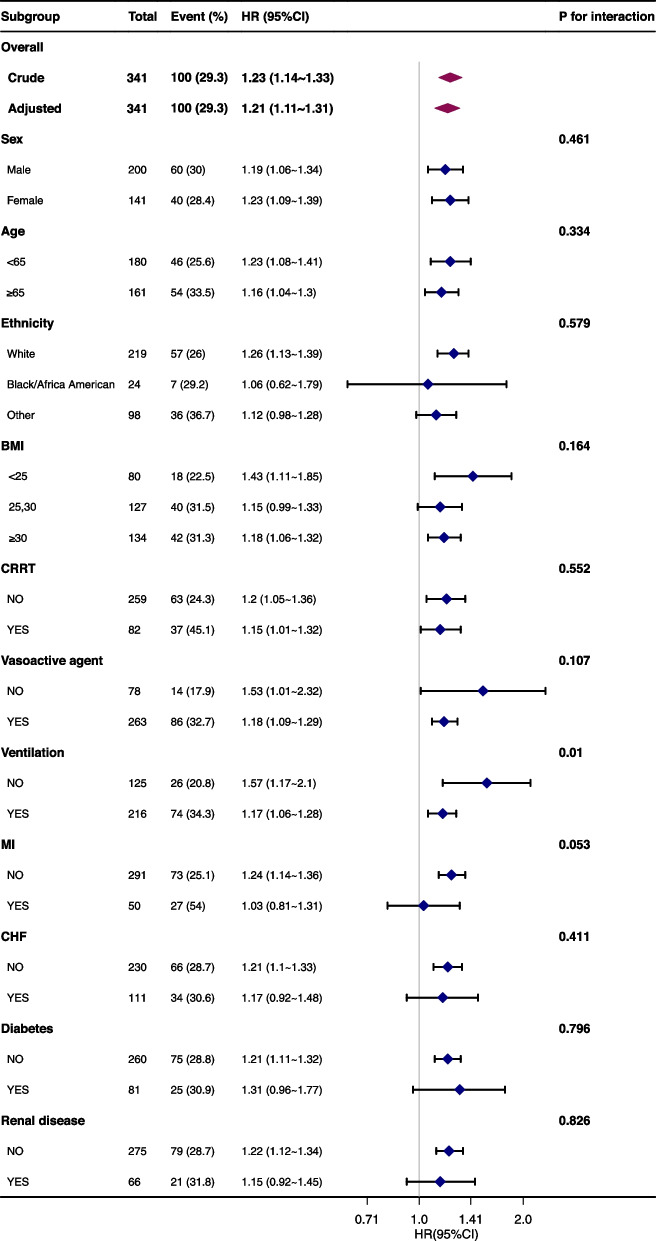
Subgroup analyses of the association between LAR and 28-day all-cause mortality. The stratification factor was not used as an adjustment variable. The small diamonds represent hazard ratios, and the horizontal line represents the 95% confidence interval. The large diamonds represent the overall HRs, whereas the outer points of the diamonds represent a 95% confidence interval

In patients with SALI, we performed a subgroup analysis to evaluate the interaction between LAR and 28-day all-cause mortality, and the results were shown in Fig. 3. In our study, there may be an interaction observed only with the use of ventilators, but the results remain stable in other subgroups (Fig. 3). Due to the limited sample size in our study, potential bias may exist, and therefore, further large-scale prospective research is needed to confirm these findings.

## Discussion

In this retrospective cohort analysis of large public datasets, we found a significant correlation between LAR and 28-day all-cause mortality in the SALI population. The Kaplan–Meier survival curve analysis indicated a higher 28-day mortality rate in the Q4 group of the LAR quartile compared to the other quartiles. Furthermore, the COX multivariate regression analysis, following adjustment for confounding factors, consistently revealed a significant rise in the risk of 28-day mortality among SALI patients with an increased LAR.

Our research finds a noteworthy association between LAR and mortality of SALI. Recent investigations have demonstrated the predictive utility of LAR in mortality prediction across diverse conditions, including Heart Failure [22], Sepsis [23], and Acute Respiratory Failure [24]. Consistent with these insights, our study highlights a distinct association between heightened LAR and mortality. Our study found a 28-day all-cause mortality rate of 29.3% in patients with SALI, consistent with other literature [25, 26] supporting the high mortality associated with SALI. Therefore, SALI is a health area that merits attention from numerous scholars. Notably, prior studies have not delved into the role of LAR in the context of SALI. Our research expands the applicability of LAR to the cohort of patients afflicted with SALI, contributing novel insights to this specific medical context.

Our investigation includes the evaluation of both lactate and albumin in the context of SALI. The results reveal that heightened lactate levels, reduced albumin levels, and an increased LAR are correlated with heightened mortality risk in individuals with SALI. This observation aligns with the emerging recognition of lactate and albumin as potential biomarkers for diverse pathological conditions. Lactate serves as an indicator of tissue hypoxia, while albumin plays a role in the redox processes associated with various diseases [27]. Notably, existing research proves a robust correlation between lactate levels and mortality in patients with suspected infections and sepsis [28]. In a study on the mortality rate of COVID-19 concerning albumin, the findings revealed a negative correlation, with an increase in mortality rate as albumin levels decrease [29]. Notably, in a cohort study, albumin emerged as an independent predictor of mortality in elderly sepsis cases [30]. Serum albumin levels can increase following the infusion of human albumin, especially when left untreated. As albumin contributes to the increase in colloid osmotic pressure, several studies have confirmed its potential therapeutic use in diseases such as sepsis and other conditions [31–33]. Consequently, our study exclusively incorporated laboratory indicators from the first day of patient admission, minimizing the potential impact of treatment and ensuring the reliability of our results. The Multivariable Cox regression analysis provided evidence of a significant correlation between LAR and the 28-day all-cause mortality rate in patients with SALI. Therefore, LAR may be an independent risk factor for all-cause mortality in SALI patients.

Through separate multivariable regression analyses for LAR, lactate, and albumin, we observed that SALI patients have a higher risk of mortality with increasing LAR (Supplementary Table 4). Studies have shown that the LAR exhibits enhanced predictive accuracy for sepsis prognosis compared to individual assessments of lactate and albumin. A prospective study found that LAR was better than lactic acid in predicting in-hospital mortality in patients with sepsis [34]. Kamran Shadvar et al. [35] conducted a study involving 151 patients with septic shock. Utilizing ROC curve analysis and logistic multivariate regression, they discovered that the LAR outperformed both lactate levels and lactate clearance rate in predicting the prognosis of septic shock patients. In a separate investigation by Cakir et al. [23], encompassing 1136 sepsis patients admitted to the ICU, the LAR emerged as a more reliable predictor of clinical outcomes in sepsis patients, as determined by the area under the ROC curve, compared to individual assessments of lactate and albumin. While these findings contribute to our understanding of the prognostic implications of LAR in sepsis treatment, they do not offer insights into SALI. Our study, however, establishes a significant correlation between LAR and the 28-day mortality rate in the context of SALI. Meanwhile, K-M survival curve analysis showed that the mortality rate in the highest quartile group was higher than that in other groups. These findings offer valuable insights into prognostic indicators for SALI, particularly the association between LAR and adverse outcomes in SALI patients.

We conducted a sensitivity analysis using diagnostic criteria for SALI from the literature that differed from those used in our study. The hazard ratio (HR) in the sensitivity analysis was 1.49 (1.13–1.96), in contrast to the main study where the HR stood at 1.21 (1.11–1.31). Consistent results confirmed a significant association between LAR and SALI. In a study investigating AST/platelet ratio and SALI, the criteria were set at total bilirubin equal to or greater than 4 mg/dl and ALT exceeding 2 times the upper limit of normal [8]. Our sensitivity analysis employed the diagnostic criteria outlined in the referenced literature. In our study, we adhered to the diagnostic standards outlined in the Surviving Sepsis Campaign guidelines [20], a criterion aligned with multiple literature on SALI. In Dou's study, although there were no indicators for coagulation function, the diagnostic criteria included higher bilirubin concentrations. Moreover, a cohort study revealed an association between elevated total bilirubin levels and adverse outcomes in patients with SALI [36]. Hence, we speculate that elevated bilirubin levels may be one of the reasons for the increased risk of SALI mortality identified in our sensitivity analysis. Through sensitivity analysis, the significant correlation between LAR and mortality in SALI patients was once again validated. Therefore, LAR can be extended as an indicator of adverse outcomes in SALI patients admitted to the ICU, offering clinicians new perspectives for consideration.

LAR emerges as a potential independent risk factor for the 28-day mortality rate in SALI patients. Through factor regression analysis and sensitivity analyses based on varying diagnostic criteria, a significant correlation between the two has been established. Subgroup analyses in our study also indicate no interaction effects when stratifying by age, gender, race, BMI, CRRT, vasoactive agent exposure, and comorbidities. Interaction effects were observed only in the subgroup using mechanical ventilation. However, due to limitations in our sample size, especially with reduced numbers after stratification, the results may exhibit bias, warranting further validation through large-scale prospective studies.

### Limitations

Our study has several limitations. First, because this was a single-center retrospective cohort study, the generalizability of our findings may be limited. Therefore, well-designed multicenter prospective studies in this field would enhance the reliability of the research results. Second, our study focused on the association between the first LAR after admission and the 28-day mortality, which is not fully applicable to the relationship between dynamic LAR and mortality. Next, we designed a dynamic monitoring index study. Third, the diagnosis of sepsis-related liver injury has different criteria, which makes the single research results not fully applicable to the population with different diagnostic criteria. However, in our study, COX multivariate regression analysis was used to analyze the sensitivity of data with different diagnostic criteria for SALI, which showed that our research results are reliable. Finally, our dataset may include patients with comorbidities such as renal disease and diabetes, which may affect lactate and albumin levels. Nevertheless, we also conducted a subgroup analysis of renal disease and diabetes, which proved the robustness of our results.

## Conclusion

In conclusion, LAR can be used as an independent risk factor for all-cause mortality in patients of SALI within 28-d of admission. As LAR increases, there is a corresponding rise in the 28-day mortality rate. However, further validation through a large-scale multicenter prospective study is required to establish its widespread applicability and objectivity.

### Supplementary Information

**Additional file 1:** **Supplementary Table 1.** Specific value and percentage of missing variable.

**Additional file 2:** **Supplementary Table 2.** After Multiple interpolations, Multivariate COX analysis between LAR level and 28-day mortality.

**Additional file 3:** **Supplementary Table 3.** Sensitive analysis: Multivariate COX analysis between LAR level and 28-day mortality.

**Additional file 4:** **Supplementary table 4.** Multivariate regression analysis of three variables.

**Additional file 5:** **Supplementary Figure**. Restricted cubic spline modeling of the association between LAR and 28-day mortality in patients with SALI. RCS restricted cubic spline; OR odds ratio; LAR lactate-to-albumin ratio; SALI sepsis-associated liver injury.

## Data Availability

The datasets generated and analyzed during the current study are available in the Medical Information Mart for Intensive Care IV (https://mimic.physionet.org/).
