# Supplementary material for: Association between lactate-to-albumin ratio and 28-days all-cause mortality in patients with sepsis-associated liver injury: a retrospective cohort study
Source: BMC Infect Dis. 2024 Jan 9;24:65. doi: 10.1186/s12879-024-08978-x (PMC10775525; doi:10.1186/s12879-024-08978-x)
Supplement: Supplementary file 1 — Additional file 1: Supplementary Table 1. Specific value and percentage of missing variable. [file 12879_2024_8978_MOESM1_ESM.docx]

**Supplementary Table 1 Specific value and percentage of missing variable**

| **Variable** | **Miss Frequency** | **Miss. percentage%** |
| --- | --- | --- |
| **BMI: Body Mass Index** | **99** | **29.0323** |
| **Calcium** | **2** | **0.5865** |
| **Fibrinogen** | **101** | **29.6188** |
| **Height** | **98** | **28.739** |
| **LDL: Low-Density Lipoprotein** | **68** | **19.9413** |
| **PTT: Activated partial thromboplastin time** | **2** | **0.5865** |
| **RBC: Red blood cell counts** | **47** | **13.783** |
| **RDW: Red blood cell distribution width** | **47** | **13.783** |
| **Temperature** | **19** | **5.5718** |
| **Weight** | **1** | **0.2933** |
