# Supplementary material for: Association between lactate-to-albumin ratio and 28-days all-cause mortality in patients with sepsis-associated liver injury: a retrospective cohort study
Source: BMC Infect Dis. 2024 Jan 9;24:65. doi: 10.1186/s12879-024-08978-x (PMC10775525; doi:10.1186/s12879-024-08978-x)
Supplement: Supplementary file 2 — Additional file 2: Supplementary Table 2. After Multiple interpolations, Multivariate COX analysis between LAR level and 28-day mortality. [file 12879_2024_8978_MOESM2_ESM.docx]

**Supplementary Table 2** After Multiple interpolations, Multivariate COX analysis between LAR level and 28-day mortality

| Variable | Crude | |  | Adjusted | |
| --- | --- | --- | --- | --- | --- |
|  | HR (95%CI) | P value |  | HR (95%CI) | P value |
| LAR | 1.23 (1.14~1.33) | <0.001 |  | 1.54 (1.23~1.94) | 0.001 |
| LAR.Q |  |  |  |  |  |
| LAR.Q1 | Ref |  |  | Ref |  |
| LAR.Q2 | 1.57 (0.8~3.07) | 0.189 |  | 5.31 (1.97~14.32) | 0.003 |
| LAR.Q3 | 1.78 (0.91~3.45) | 0.094 |  | 3.5 (1.14~10.72) | 0.04 |
| LAR.Q4 | 3.63 (1.98~6.67) | <0.001 |  | 10.28 (2.83~37.33) | 0.002 |
