# Supplementary material for: Association between lactate-to-albumin ratio and 28-days all-cause mortality in patients with sepsis-associated liver injury: a retrospective cohort study
Source: BMC Infect Dis. 2024 Jan 9;24:65. doi: 10.1186/s12879-024-08978-x (PMC10775525; doi:10.1186/s12879-024-08978-x)
Supplement: Supplementary file 3 — Additional file 3: Supplementary Table 3. Sensitive analysis: Multivariate COX analysis between LAR level and 28-day mortality. [file 12879_2024_8978_MOESM3_ESM.docx]

**Supplementary Table 3** Sensitive analysis: Multivariate COX analysis between LAR level and 28-day mortality

| Variable | N total | N event_% | Non-adjusted | |  | Model 1 | |  | Model 2 | |  | Model 3 | |
| --- | --- | --- | --- | --- | --- | --- | --- | --- | --- | --- | --- | --- | --- |
|  |  |  | HR (95%CI) | P value |  | HR (95%CI) | P value |  | HR (95%CI) | P value |  | HR (95%CI) | P value |
| LAR | 199 | 61 (30.7) | 1.28 (1.18~1.39) | <0.001 |  | 1.28 (1.18~1.39) | <0.001 |  | 1.18 (1.02~1.37) | 0.023 |  | 1.49 (1.13~1.96) | 0.004 |
|  |  |  |  |  |  |  |  |  |  |  |  |  |  |
| LAR |  |  |  |  |  |  |  |  |  |  |  |  |  |
| LAR.Q1 | 50 | 8 (16) | 1(Ref) |  |  | 1(Ref) |  |  | 1(Ref) |  |  | 1(Ref) |  |
| LAR.Q2 | 49 | 7 (14.3) | 0.91 (0.33~2.51) | 0.858 |  | 0.93 (0.34~2.58) | 0.894 |  | 1.19 (0.34~4.19) | 0.783 |  | 12.76 (0.98~166.65) | 0.052 |
| LAR.Q3 | 50 | 18 (36) | 2.49 (1.08~5.73) | 0.032 |  | 2.94 (1.26~6.83) | 0.012 |  | 3.63 (1.23~10.75) | 0.02 |  | 18.67 (1.73~201.72) | 0.016 |
| LAR.Q4 | 50 | 28 (56) | 5.08 (2.31~11.17) | <0.001 |  | 5.67 (2.57~12.5) | <0.001 |  | 5.42 (1.6~18.37) | 0.007 |  | 47.82 (3.6~634.58) | 0.003 |
| P for trend |  |  |  | <0.001 |  |  | <0.001 |  |  | 0.002 |  |  | 0.003 |

Notes: LAR.Q1(≤0.76); LAR.Q2(0.77-1.49); LAR.Q3(1.50-3); LAR.Q4(>3).

MODEL 1: sex, age

MODEL 2: sex, age, Ethnicity, BMI, temperature, insurance, and admission type.

MODEL 3: sex, age, Ethnicity, BMI, insurance, admission type, temperature, WBC, platelet, calcium, potassium, fibrinogen, PT, PTT, ALP, TB, and PO_2_.
