# Supplementary material for: Association between lactate-to-albumin ratio and 28-days all-cause mortality in patients with sepsis-associated liver injury: a retrospective cohort study
Source: BMC Infect Dis. 2024 Jan 9;24:65. doi: 10.1186/s12879-024-08978-x (PMC10775525; doi:10.1186/s12879-024-08978-x)
Supplement: Supplementary file 4 — Additional file 4: Supplementary table 4. Multivariate regression analysis of three variables. [file 12879_2024_8978_MOESM4_ESM.docx]

**Supplementary table 4** Multivariate regression analysis of three variables

| Variable | Crude | |  | MODEL 1 | |  | MODEL 2 | |  | MODEL 3 | |
| --- | --- | --- | --- | --- | --- | --- | --- | --- | --- | --- | --- |
|  | HR(95%CI) | P |  | HR(95%CI) | P |  | HR(95%CI) | P |  | HR(95%CI) | P |
| LAR | 1.23 (1.14~1.33) | <0.001 |  | 1.24 (1.15~1.34) | <0.001 |  | 1.19 (1.1~1.29) | <0.001 |  | 1.21 (1.11~1.31) | <0.001 |
| lactate | 1.14 (1.08~1.19) | <0.001 |  | 1.14 (1.09~1.19) | <0.001 |  | 1.12 (1.06~1.17) | <0.001 |  | 1.14 (1.08~1.2) | <0.001 |
| albumin | 0.75 (0.55~1.02) | 0.069 |  | 0.73 (0.54~1) | 0.051 |  | 0.79 (0.58~1.09) | 0.154 |  | 0.81 (0.59~1.12) | 0.199 |

MODEL 1: sex, age

MODEL 2: sex, age, MBP, SpO_2_

MODEL 3: sex, age, MBP, SpO2, BUN, RDW, PTT, Vasoactive agent
