# Supplementary figures and images for: Association between lactate-to-albumin ratio and 28-days all-cause mortality in patients with sepsis-associated liver injury: a retrospective cohort study
Source: BMC Infect Dis. 2024 Jan 9;24:65. doi: 10.1186/s12879-024-08978-x (PMC10775525; doi:10.1186/s12879-024-08978-x)

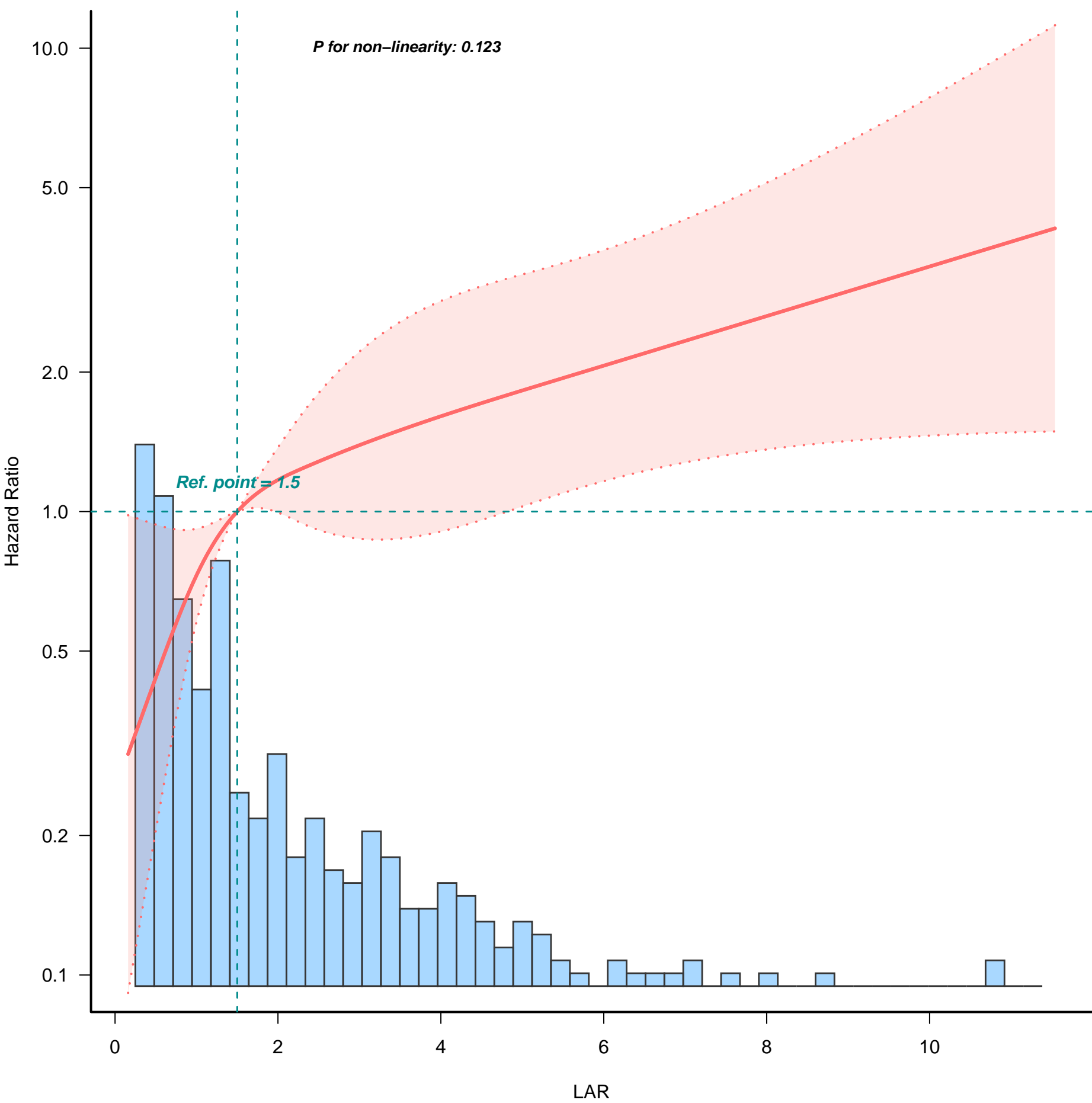

Supplement: Supplementary file 5 — Additional file 5: Supplementary Figure. Restricted cubic spline modeling of the association between LAR and 28-day mortality in patients with SALI. RCS restricted cubic spline; OR odds ratio; LAR lactate-to-albumin ratio; SALI sepsis-associated liver injury. [file 12879_2024_8978_MOESM5_ESM.pdf]
